# Altruism – the enigma of self-interest

**DOI:** 10.1186/1753-6561-5-S1-L12

**Published:** 2011-01-10

**Authors:** Thomas Sze Tong Chan

**Affiliations:** 1 World Vision, Hong Kong, Hong Kong SAR

## 

The definition of altruism will be presented, highlighting the enigma behind this phenomenon in today’s highly competitive world of market economy.

The growing challenges of hunger and poverty plague the world even as we entered the new millennium. As the gap between the rich and the poor widens, there is also a trend showing the increase of charitable acts and a growing interest in altruistic activities.

Hong Kong, where the East meets the West, has developed into a community that is highly competitive and market-oriented. Yet its citizens, while famous for being hard-driven and target-oriented, have also nurtured a charitable spirit that is widespread and deep-rooted. Most, if not all, of her citizen regularly engage in charitable donations and sacrificial services. Each year, the community here donates an amount that, on a per capita basis, ranks amount the top of the world. Data from donor profiling by one of the larger fundraising charities provide clues as to why people give and how they choose their favorite charities.

While donating money was the starting point of charitable work, current trend showed that more and more people now choose to donate their time serving the needy, by volunteering and even working fulltime with charities of their choice.

The author recounts his 14-year experience of fulltime service with an international charitable organization in Mainland China and East Asia, and discusses what motivated many of his colleagues to serve in impoverished regions of Asia. Foregoing the comfort and luxuries of modern world, young professional people with high education background chose to use their professional skills to serve the needy rather than making money for themselves. The motivation behind their decisions is discussed.

Evidence point to the fact that instead of being selfless, most donors and workers choose to serve because of a conviction that serving others bring satisfaction and / or reward to themselves. This concept of rewards is of course not in monetary terms. Alternative worldviews are common among workers in charitable organizations. The basis of these worldviews will be discussed.

